# Eutectic Phase Characterization and Mechanical Properties of Al-Cu Alloy Ingot Solidified with Ultrasonic Treatment

**DOI:** 10.3390/ma15031067

**Published:** 2022-01-29

**Authors:** Ruiqing Li, Fang Dong, Yun Zhang, Pinghu Chen, Xiaoqian Li

**Affiliations:** 1 Light Alloy Research Institute, State Key Laboratory of High Performance Complex Manufacturing, Central South University, Changsha 410012, China; liruiqing@csu.edu.cn (R.L.); meel@csu.edu.cn (X.L.); 2 College of Mechanical and Electrical Engineering, Central South University, Changsha 410083, China; yun_zhang66@163.com; 3 College of Mechatronics & Control Engineering, Additive Manufacturing Institute, Shenzhen University, Shenzhen 518060, China; chenpinghu1986@163.com

**Keywords:** ultrasonic, aluminum alloy, simulation, eutectic phase, quantitative characterization

## Abstract

An Al-Cu alloy ingot was produced with the application of ultrasonic melt treatment. The effects of ultrasonication on the grain structure, eutectic phase, solution, and tensile properties of the alloy were analyzed. The volume and distribution of the eutectic phase were quantitatively evaluated based on stereological theory. The results are as follows: The grain-refinement efficiency at the center, 1/2 radius and edge of the ingot is 33.99%, 45.2% and 41.68%, respectively, under the action of an ultrasonic field. Ultrasonics improves the solid solubility of the Al-Cu alloy element, in which the solid solubility of Cu increases from 0.85% to 1.42%. The ultrasonic field improves the dispersion degree of the eutectic phase and reduces the volume fraction and eutectic phase number per unit volume. The mechanical properties of the Al-Cu alloy were improved by an ultrasonic field.

## 1. Introduction

Aluminum alloys have been widely used in the aerospace, automotive and other fields due to their light weight, high strength, and other features [1,2]. Among them, aerospace ring/cylindrical structural parts are generally obtained by deformation processing of aluminum-copper alloy ingots. Therefore, the quality of the ingots is critical to the formation of ring/cylindrical parts. However, the quality of aluminum alloy structural parts is seriously restricted by defects, such as coarse microstructure, severe segregation, porosity inclusion, and even cracking in large aluminum alloy ingots [3,4]. In particular, the most important reason for the unstable mechanical properties of the final ring/cylindrical member is the enrichment of residual eutectic phases in the component. When the residual eutectic phase is formed in large quantities at the grain boundary, the stress concentration is caused by the difference in the material properties between the residual eutectic phase and the matrix, which leads to the initiation of microcracks at the grain boundary [5]. The agglomerated eutectic phases are most likely to become crack nucleation sites during the service of the components in the later stage and lead to crack propagation [6]. At the same time, the ductility of the aluminum alloy is greatly reduced by the coarse and hard eutectic phases [7]. Therefore, it is very important to quantitatively detect and evaluate the eutectic phase of an ingot. However, at present, the quality-inspection system of aluminum alloy is not excellent because it only includes grain size, alloy composition, porosity, pores, coarse inclusions, and other indicators, and no eutectic phases are mentioned.

The solidification process of aluminum alloys includes a series of behaviors, such as heat and mass transfer, melt nucleation, and crystal growth [8]. Ultrasonic vibration treatment of alloy melts is a physical treatment technique that has gained widespread support from the scientific community in recent years [9,10]. When an ultrasonic field is applied to the aluminum alloy melt, the environment, as well as the physical and chemical conditions, of the solidification process of the aluminum alloy melt will be changed, and the structure and properties of the ingot will be altered accordingly [11]. Some research on the ultrasonic treatment of metal melts has been published, mainly by Eskin [12] and Eskin et al. [13]. They found that ultrasound can significantly improve the morphology of the structure, refine the grains, and degas aluminum alloy ingots due to its special effects, such as cavitation and acoustic streaming.

In casting studies, ultrasonically stirred samples have finer microstructures [14]. Ultrasonic treatment can effectively control the morphology and size of aluminum grains [15]. Ultrasonic vibration has been proven to be effective in controlling the columnar dendritic structure, reducing the size of equiaxed grains, and, under certain conditions, producing spherical, nondendritic grains [16]. Li et al. [17,18,19] investigated the mechanisms of the ultrasonic field on Al alloy during the direct-chill (DC) casting process and proposed the mechanism of ultrasonic heterogeneous activation and the crystal resonance effect. In previous studies of large industrial ingots, an ultrasonic generator was employed with frequencies, powers, and peak-to-peak amplitudes of 20 ± 1 kHz, 0.5–1 kW, and 20 ± 1.0 µm, respectively. [20,21] Due to the small ingot size in this study, the power of ultrasound was 200 W.

In addition, high-quality 7XXX and 2XXX aluminum round/flat ingots with different sizes were manufactured, and successful application promoted the development of the manufacturing field of high-performance, light-alloy, large, and complex structural components for aerospace applications. Previous studies found that the coarse crystalline phase seriously reduced the plasticity of the material [22,23].

The characterization of the crystalline phase in the ingot is therefore very important. However, no accurate quantitative analysis study of the eutectic phase has been reported thus far.

In the present study, ultrasound-assisted solidification tests of small Al-Cu alloy ingots were carried out under laboratory conditions, and the effects of an ultrasonic field on the grain structure, eutectic phase, and intragranular solid solution of alloy elements were studied systematically. To quantitatively measure the content of the eutectic phase, this paper proposes a quantitative measurement method of the eutectic phase based on the image-processing function of MATLAB 9.0 software, the backscattering diagram of the crystal phase, and the stereology formula. Meanwhile, the volume of the eutectic phase in the Al-Cu conventional ingot and ultrasonic ingot were compared to illustrate the effect mechanisms of ultrasound on the microstructure transformation during solidification.

## 2. Materials and Methods

### 2.1. Experimental Equipment

**Figure 1:**
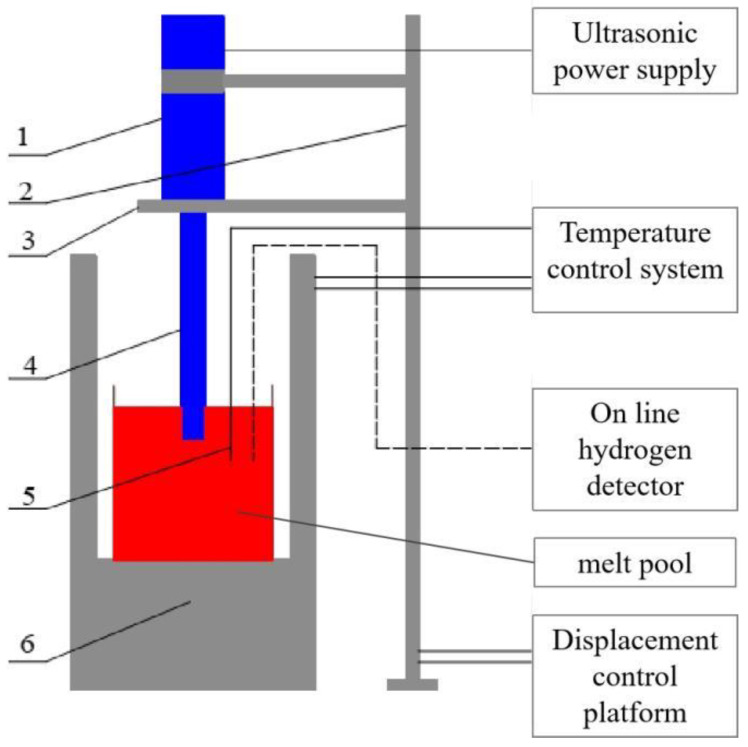
Schematic diagram of the Al-Cu alloy experimental device for ultrasonic-assisted casting. 1: ultrasonic transducer; 2: positioning support flange; 3: displacement control device; 4: ultrasonic horn; 5: thermocouple; 6: resistance-wire heating furnace.

The experimental equipment used in this study is as follows: (1) ultrasonic system, which is used to output vibration, including ultrasonic power supply (200 W for output power and 20 kHz for output frequency), PZT piezoelectric ceramic transducer, #45 steel horn, and titanium alloy radiation rod; (2) temperature control and detection system, including resistance furnace, which is used to heat raw material, and thermocouple temperature sensor, which is used to detect temperature; (3) auxiliary equipment, including a graphite clay crucible (external dimension: diameter is 206 mm × 220 mm, and thickness is 19 mm), displacement control platform, timer, tray balance, and online hydrogen detector, which is used during casting. The whole experimental casting device of the crucible is shown in Figure 1.

### 2.2. Melting and Ultrasonic Treatment Process

The prepared Al-Cu alloys taken from industrial ingots were put into a graphite crucible for smelting at 750 °C, followed by slagging off. The radiation rod was preheated before the experiment to avoid shock chilling. Ultrasonic treatment was carried out when the melt temperature reached 700 °C. The ultrasonic rod was located in the center of the ingot, with an insertion depth of 35 mm, and the displacement system was used to fix the ultrasonic position. After application of ultrasound for 15 min, the ultrasonic rod was pulled out slowly and stably, and the aluminum melt was poured into a crucible to be cooled in water. Meanwhile, a conventional ingot cast under the same conditions without ultrasonic treatment was used for comparison. The main chemical compositions of the Al-Cu alloy ingot are shown in Table 1.

### 2.3. Sample Preparation and Testing

**Figure 2:**
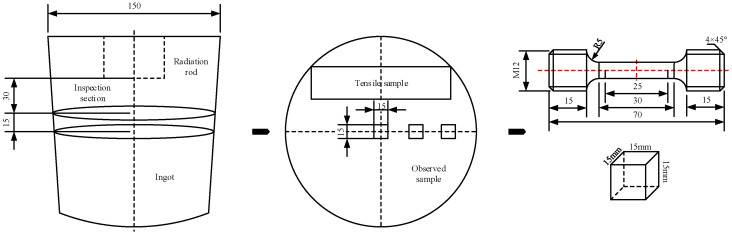
Schematic diagram of test-sample preparation.

Samples were cut from the two ingots with the method shown in Figure 2. Testing samples were sectioned 30 mm away from the end surface of the radiation rod. Three cube samples with a size of 15 mm were cut from the center, 1/2 radius, and edge along the radius of the ingot. Subsequently, the metallographic samples were mechanically ground and polished, followed by etching with Keller’s solution (1% HF, 1.5% HCl, and 2.5 vol.% HNO3). The eutectic phase of the samples was observed by scanning electron microscopy (TESCAN^®^ MIRA3, Brno, Czech Republic). The relative solid solubility of elements in a grain was measured by an energy-dispersive spectrometer (Inca^®^ Energy X-Max20, London, UK). The grain-structure characteristics were observed by a Leica^®^ metallographic microscope (56XC, Vizsla, Germany) after standard metallographic sample preparation. Tensile tests were conducted on an Instron^®^ 3369 mechanical testing machine with a tensile speed of 2 mm/min.

### 2.4. The Method of Quantitative Detection for Eutectic Phase

Quantitative analysis of the eutectic phase is important for detecting the quality of ingots. Eutectic-morphology detection results are essential for performing quantitative analysis. The morphology and distribution of the eutectic phase can be observed by scanning electron microscopy (SEM), and the quantitative relationship between the eutectic phase and ingot quality can be established. However, quantitative analysis of the eutectic phase usually requires mathematical methods and analysis by measuring various characteristic parameters of the eutectic phase in the backscatter diagram.

In this work, the volume and distribution of the eutectic phase in two ingots are quantitatively characterized by three parameters, including *V_V_*, which is the symbol of an integral number of eutectic phases; *P_V_*, which is the symbol of the number of eutectic phases per unit volume; and λ, which is the mean distance between eutectic-phase particles. λ is used to quantitatively evaluate the dispersion degree of the eutectic phase in the ingot. The mean free path is defined as the average distance of a particle in an object traveling when it collides with other particles. That is, the more eutectic phases there are per unit volume, the more crowded and frequent the collisions and the smaller the free path will be. In contrast, the larger the free path, the greater the dispersion degree of the eutectic phase. In this work, the backscattering images of alloy ingots were processed and counted by the image-processing tool of MATLAB software based on stereology principles [24,25].

First, the backscattering SEM image was read by MATLAB software and then preprocessed by an image-processing tool kit (image gray processing, enhancement processing, and filtering processing). The image was morphologically processed, including expansion operation, corrosion operation, open operation, and closed operation. Then, the eutectic phase characteristics of the image were extracted after edge detection and area statistics. The eutectic phase can be conveniently and efficiently quantitatively analyzed by the stereological formula [26,27].

**Figure 3:**
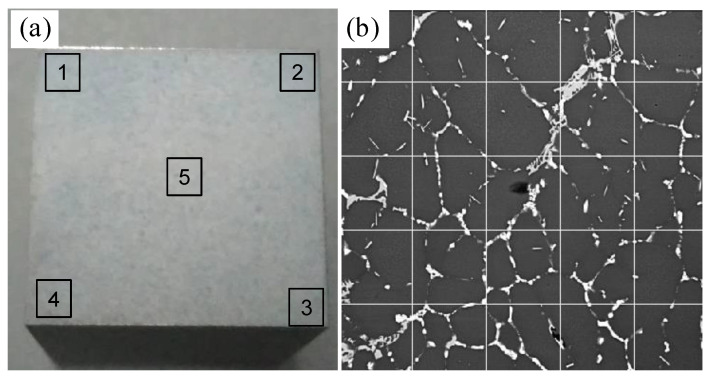
Measurement of the eutectic phase by the cross-section method and the linear intercept method. (**a**) Schematic diagram of selected areas; (**b**) schematic diagram of division of the linear intercept method of backscatter image.

It can be seen from the backscatter image of the sample that the distribution of the eutectic phase in the ingot was dispersed on the whole sample surface. Therefore, the statistics of eutectic phases that relied only on a single measuring line had a large error. As shown in Figure 3, multiple horizontal lines and vertical lines were selected as measuring lines to measure the backscatter image at a magnification of 500 times. That is, eight measuring lines were prepared in five areas of the sample (600 × 600 μm^2^), including four horizontal lines and four vertical lines. The number of points where the unit-length test line intersects the eutectic phase in the backscatter diagram was recorded as *P_L_*. Similarly, the backscatter image was taken in five random areas of each sample, as shown in Figure 3a, and each backscatter image was divided by the linear intercept method, as shown in Figure 3b. $\bar{P_{L}}$, which is the average value of *P_L_* of the five backscatter sketches, was taken to represent the number of intersections of the unit length of measuring lines and the eutectic phase. After the $\bar{P_{L}}$ of the sample was determined, it was necessary to count *P_A_*, which represents the number of eutectic phases per unit area tested in the whole backscatter image. In the same way, the average value, $\bar{P_{A}}$, of *P_A_* of the five backscatter sketches was taken, and then the stereological equation, $P_{V}=2\bar{P_{A}}\bar{P_{L}}$, was used to obtain *P_V_*, which is the number of intersections of eutectic phases in the unit test volume of the sample.

## 3. Results

### 3.1. Grain Structure

**Figure 4:**
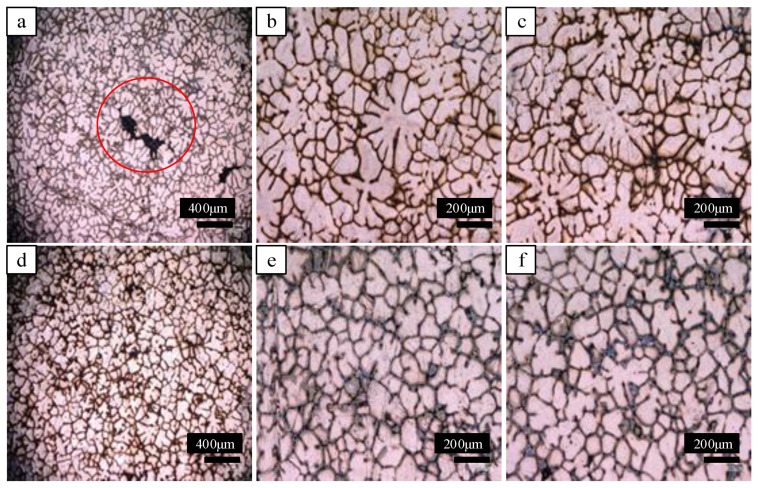
Grain structure of conventional and ultrasonic ingots: (**a**) edge of conventional ingot; (**b**) 1/2 radius of conventional ingot; (**c**) center of conventional ingot; (**d**) edge of ultrasonic ingot; (**e**) 1/2 radius of ultrasonic ingot; (**f**) center of ultrasonic ingot.

Figure 4 shows the grain structures. The grains of conventional ingots are mostly coarse dendrites, with developed dendrites and many defects appearing in the center, as shown in the red circle area in Figure 4a. However, the grains of the ultrasonic ingot are fine, and many grains are equiaxed, as shown in Figure 4d,e. The grain structure of the ingot is obviously refined by ultrasonication, and defects, such as pores and porosity in the ingot structure, are reduced by ultrasonication. The cavitation and acoustic-streaming effect of ultrasonication can improve the compactness of the ingot structure by refining the grain structure of the ingot and reducing the defects [11,28,29].

The grain size at different positions of the ingot was measured. The average grain size at the center, 1/2 radius, and edge of the conventional ingot was 253 μm, 300 μm, and 280 μm, respectively. However, the average grain sizes of the three locations of the ultrasonic ingot were 167 μm, 164 μm, and 163 μm. The grain size of the whole cross section of the ultrasonic ingot was refined and more uniform.

To more intuitively describe the effect of ultrasonication on grain refinement, the refinement efficiency of the grains was calculated by the following formula:

(1) $$\eta_{i}=\frac{D_{i}-d_{i}}{D_{i}}$$

where $\eta_{i}$ is the grain refinement rate; $D_{i}$ is the average grain size at a certain position of the conventional ingot; and $d_{i}$ is the average grain size of the ultrasonic ingot.

According to Formula (1), the refinement ratios of ultrasonication at the center, 1/2 radius, and edge of the crucible ingot were 33.99%, 45.2%, and 41.68%, respectively. The grains across the whole ingot were refined by ultrasound to different degrees.

### 3.2. Eutectic Morphology and Quantitative Analysis

**Figure 5:**
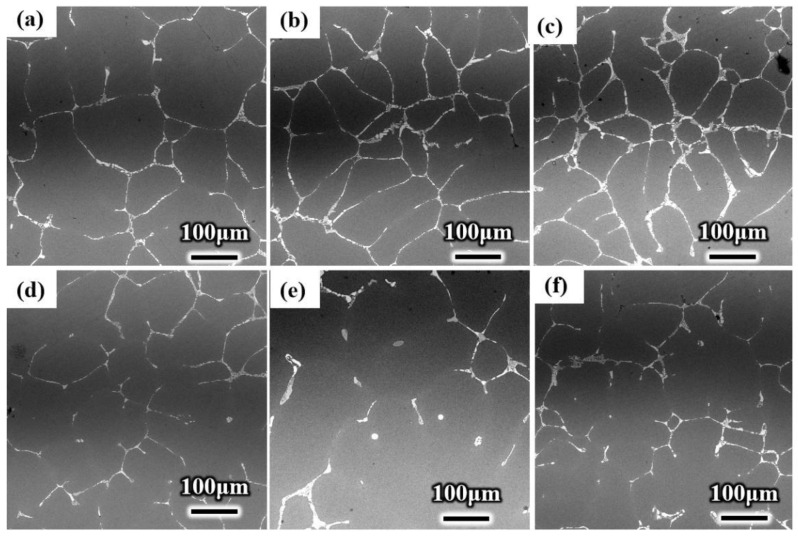
The eutectic phase distribution of the ultrasonic ingot and conventional ingot. (**a**) Edge of conventional ingot; (**b**) 1/2 radius of conventional ingot; (**c**) center of conventional ingot; (**d**) edge of ultrasonic ingot; (**e**) 1/2 radius of ultrasonic ingot; (**f**) center of ultrasonic ingot.

Figure 5 shows the eutectic structure of the two ingots. White with grid-like eutectic structures can be seen on the grain boundaries. The same variation tendency in eutectic distribution can be found along the radical direction of the two ingots. At the edge of the ingot, the eutectic structure is intermittent and strips. At the 1/2 radius of the ingot, the eutectic phase was connected into a grid and became continuous, while at the center of the ingot, the eutectic phase turned into agglomerated chunks with high continuity. However, at the three representative positions, the cluster degree of the eutectic phase was obviously reduced under ultrasound.

**Figure 6:**
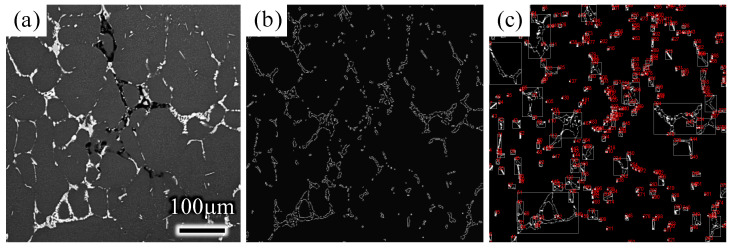
Diagram of inspection. (**a**) Original backscattering SEM image; (**b**) Image after image-marginalization processing; (**c**) image segmentation.

As shown in Figure 6, MATLAB was used to address the contrast of the backscattering of SEM. The volume fraction of the eutectic phase, the number of eutectic phases per unit volume, and the average distance between eutectic phases can be calculated by image processing and stereology theory. Table 2 shows the comparison results of the conventional ingot and ultrasonic ingot, where a and b indicate the conventional ingot and ultrasonic ingot, respectively, and samples 1, 2, and 3 represent the center, 1/2 radius, and edge of the ingot, respectively. Compared with conventional ingots, the volume fraction of the eutectic phase and the number of eutectic phases per unit volume of ultrasonic ingots at the three locations were significantly lower, and the average spacing between eutectic phases increased in ultrasonic ingots.

It can be seen from the results that the coarsening and agglomeration of the eutectic phase were more substantial at the center and 1/2 radius. The average distance between eutectic-phase particles in the center and 1/2 radius of the conventional ingot was 75 μm and 62 μm, respectively, but it was increased to 91 μm and 93 μm, respectively, after ultrasonic addition, an increase of 32% and 40%, respectively. However, due to the direct effect of cooling water at the edge of the ingot, solidification was faster. Thus, the eutectic phase was relatively dispersed, and the improvement effect of ultrasonication was smaller, only increasing by 14%.

### 3.3. Relative Intragranular Cu Content

Differences were also found in the intragranular solid solubility of solute elements in Al-Cu alloy after ultrasonic treatment. More alloying elements, which dissolve in the aluminium alloy matrix, play a role in solution strengthening for ingot materials. The mechanical properties of the ingot structure can be improved with increasing intragranular solid solubility of alloying elements in the aluminum matrix. The mass fraction of solute elements in the Al-Cu alloy was detected in the two groups of ingots. The contents of solute elements in the grains of the conventional ingot and ultrasonic ingot were scanned by an EDS spectrum analyser.

The mass fraction of the main solute elements, Cu, Mn, Mg, and Si, in the Al-Cu alloy was obtained by scanning the selected region. The average mass fraction of solute elements in this region was taken as the solid solubility of the elements in grain. The test results are shown in Table 3.

Table 3 shows that the solid solubility of each solute element in the ultrasonic ingot was improved compared with that of the conventional ingot. The solid solubility of Cu increased from 0.85% to 1.42%, which increased by 67.05% with ultrasonic treatment; the solid solubility of Mn increased from 0.36% to 0.78%, with an increase rate of 116.67%; the solid solubility of Mg increased from 1.03% to 1.18%, which was increased by 14.56%; and the solid solubility of Si increased from 0.07% to 0.28%, with an increase rate of 300%. The contents of the four alloying elements, Cu, Mg, Mn, and Si, in the Al matrix generally increased after ultrasonic treatment.

### 3.4. Mechanical Properties of Ultrasonic Ingots and Conventional Ingots

When the Al-Cu alloy material is stretched, fracture opening occurs in the weakest part of the sample, and in these parts, the grain structure is often inhomogeneous, and there will be porosity defects in the crack-forming channel, so the tensile property of the sample with fine and uniform grains and fewer porosity defects is better; otherwise, it is worse. Therefore, the mechanical tensile properties of the ultrasonic ingot and conventional ingot were tested to explore the influence of the ultrasonic field on the grain structure and porosity defects of the ingot, and the fracture of tensile samples was analyzed by SEM. The tensile test results are shown in Table 4.

The tensile strength of all three samples was improved. The average ultimate tensile strength of the ultrasonic ingot was 176.20 MPa, an increase of 8.61% after the ultrasonic field was added.

**Figure 7:**
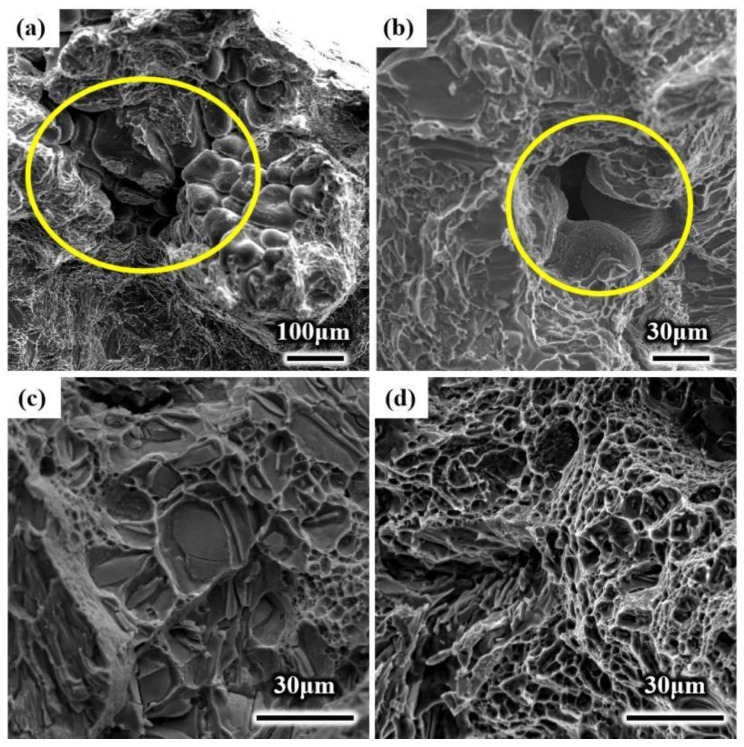
Fracture morphology of (**a**,**c**) conventional ingot and (**b**,**d**) ultrasonic ingot.

The tensile fracture surfaces of the two kinds of ingots are shown in Figure 7. Dimples can be observed on the surface of the two kinds of fracture surfaces, and defects, such as impurities, pores, casting shrinkage, and oxide film, were also distributed [24]. However, the difference between the ultrasonic and conventional tensile samples was that there was more casting shrinkage on the fracture surface of the conventional tensile samples, and the size of the shrinkage was also different. The shrinkage cavity of the conventional sample is shown in the yellow circle area in Figure 7a; the size of the shrinkage cavity was large, with a maximum of 150~200 μm. Compared with conventional samples, the size of the shrinkage cavity of the ultrasonic sample was smaller, and the size of the shrinkage cavity was only approximately 40 μm, as shown by the yellow circle in Figure 7b. The deterioration of the mechanical properties of the tensile samples was caused by the greater casting shrinkage cavity because the effective area of the external load was reduced by the increase in the shrinkage cavity, thereby increasing the possibility of sample fracture.

The dimple morphology of the ingot is shown in Figure 7c,d. Compared with that of the conventional ingot, as shown in Figure 7c, the number of dimples was larger, the size of dimples was smaller, and the depth was deeper, indicating that the plasticity of the ultrasonic ingot sample was better. Compared with the elongation rate in Table 3, the elongation rate of the ultrasonic ingot sample was generally higher, with an average value of 2.3%, compared with 1.84% of the conventional sample. When ultrasonication was added to the casting, the two-phase particles of the material could be dispersed uniformly by acoustic streaming and cavitation. Therefore, the dimple strips of ultrasonic samples were more uniform, and the plasticity of the material could be improved effectively [4,24].

**Figure 8:**
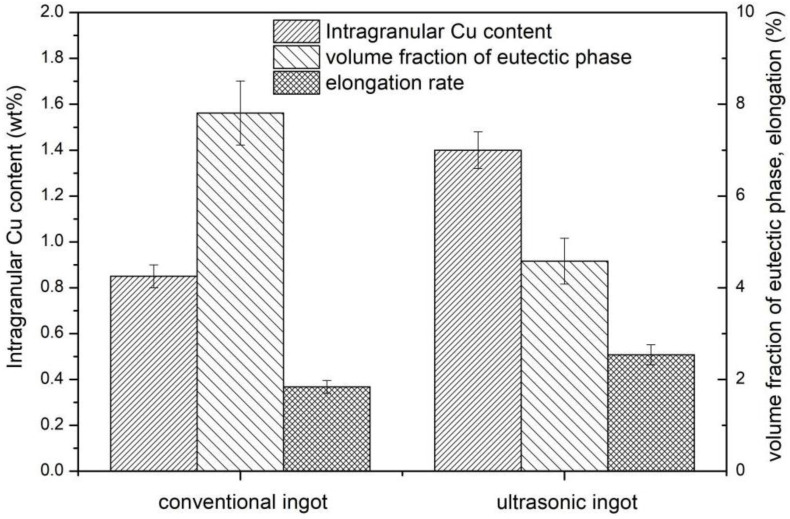
Relationship of intragranular Cu content, volume fraction of eutectic phase, and elongation rate of conventional ingot and ultrasonic ingot.

Figure 8 shows the relationship between the Cu content, volume fraction, and elongation of the common ingot and ultrasonic ingot. The higher Cu content and lower volume fraction of the intergranular eutectic structure in the ultrasonic ingot indicate that a more solid solution of Cu elements into the matrix reduces the formation of a coarse eutectic structure between grains and enhances the strengthening of the solid solution. Therefore, the mechanical properties show that the strength and elongation of the ultrasonic ingot are improved.

## 4. Discussion

### Effect of Ultrasound on Microstructural Evolution and Solute Distribution

To intuitively illustrate the effect of ultrasound on the evolution of microstructure and solute distribution, a simulation of ultrasonic solidification was also carried out [30]. According to the steady-state analysis of ultrasonic casting, the treatment of the temperature boundary has the following conditions:

(1) Entrance: the entrance is set at 973 K.

(2) Free surface: the temperature is 298 K, which contacts air, and the heat transfer coefficient is set as 50 W/m^2^·K.

(3) Wall: the inner surface of the crucible in contact with the Al melt is set as the contact surface, the outer surface of the crucible is in contact with air, the heat transfer coefficient is set to 50 W/m^2^·K, the velocity component perpendicular to the wall is 0, and the regional wall-boundary conditions are set to no-slip wall-boundary conditions.

**Figure 9:**
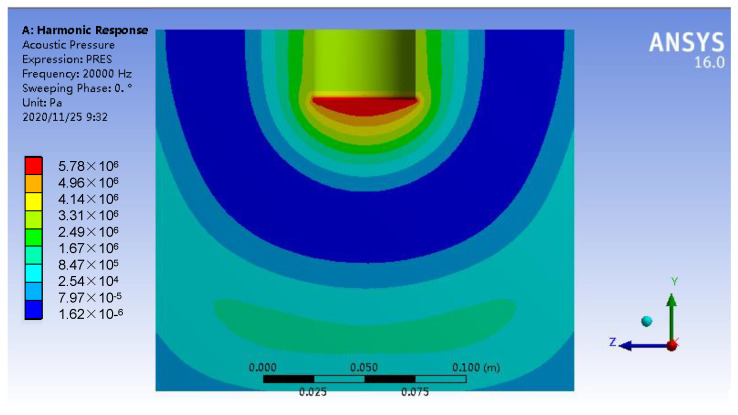
Ultrasonic sound-pressure field distribution.

Figure 9 shows a simulation diagram of the ultrasonic sound-pressure distribution in the aluminum melt. It can be seen from the diagram that the sound-pressure intensity is the largest below the end face of the ultrasonic radiation rod, up to 5.78 MPa, then decreasing down the axis of the radiation rod first, finally increasing alternately.

**Figure 10:**
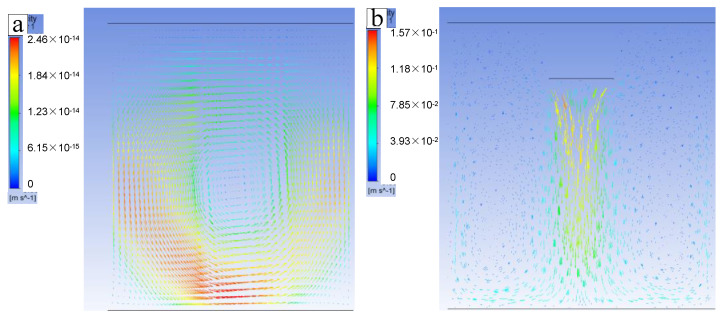
Flow patterns and velocity of the melts under (**a**) no ultrasonication and (**b**) ultrasonication.

The distribution of the flow field in the melt state is shown in Figure 10. Comparing the results of the flow-field distribution, it can be seen that the ingot, which was not ultrasonically applied, had almost no flow. However, after applying ultrasonication, the flow velocity in the molten pool is obviously increased. Figure 10 shows that the liquid flow impacts the solidification front, and the dendrites can be broken by the huge acoustic flow, which makes the broken dendrite flow [31]. In this way, the effective nucleate sites are greatly increased, and the grains are therefore refined. In addition, the solidification front was continuously scoured by ultrasonic flow, which led to the redistribution of the solute. As a result, the enrichment of solute between dendrites is reduced, and more solute is dissolved into the Al matrix.

**Figure 11:**
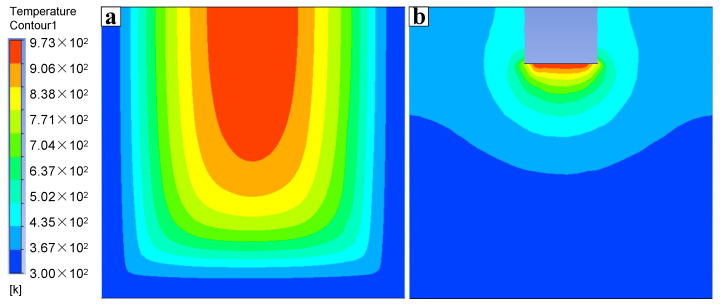
Comparison of temperature distribution between (**a**) no ultrasonication and (**b**) ultrasonication.

Figure 11 compares the temperature distribution in the crucible between no ultrasonication and ultrasonication for a period of time. It can be seen from the figure that under the same cooling conditions in the casting secondary cooling zone, when ultrasonication was not applied, the ingot center still maintained a high temperature, and the high-temperature area was large, which indicates a low cooling rate in the molten pool [32]. After introducing ultrasound, the high-temperature area in the ingot center is mainly concentrated in the first cooling zone and the upper end of the secondary cooling zone, which indicates that a large amount of heat in the Al melt is taken away from the ingot surface through the first cooling zone, and cooling water sprays on the ingot surface. The movement of the solute in the liquid and slurry regions is accelerated by the ultrasonic flow, which promotes the heat transfer of the solution at the front of solidification, makes the heat in the core transfer more quickly, and increases the cooling rate. It can be seen from the figure that the area of the high-temperature liquid phase (red part) is obviously reduced by ultrasonication, which greatly increases the nucleation area and promotes the nucleation rate. The temperature gradient in the liquid cavity is reduced by the turbulent effect of ultrasonication, which improves the temperature distribution in the center of the ingot and promotes the simultaneous nucleation of the crystal grains. Moreover, the growth rate of crystal grains is almost the same in all directions because of the uniform temperature, thus reducing the generation of dendritic crystals.

**Figure 12:**
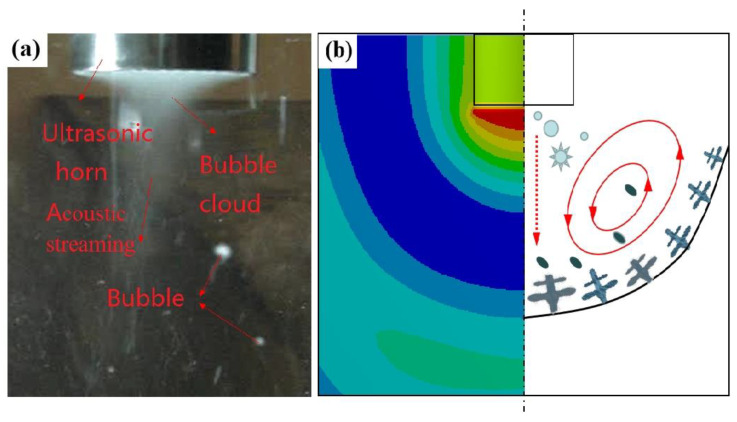
(**a**) Diagram of ultrasonic vibration in water, (**b**) mechanism diagram of ultrasonic acting on the solidification process.

Figure 12 shows ultrasonic vibration in water and mechanism diagram of ultrasonic acting on the solidification process. High temperature and high pressure generated by ultrasonic cavitation can clean the second-phase particles, reduce the wetting angle of the solid–liquid interface of the second-phase particles, promote heterogeneous nucleation, increase the nucleation rate, and refine the ingot grains. At the same time, the acoustic-streaming effect interrupted the dendrite arm, broke the dendrites, made the ingot change from dendrite to fine equiaxed crystal, and reduced the gap between grains and the chain-like second phase between grains. The eutectic phase was divided into branches and distributed intermittently. Therefore, the agglomeration of the eutectic phase was inhibited by the acoustic-streaming effect.

Based on the Hall–Petch theory, the relationship between grain size and yield strength can be expressed as follows [33,34]:

(2) $$\sigma_{\mathrm{YS}}{=\sigma }_{0}{+\mathrm{Kd}}^{-1/2}$$

where $\sigma_{\mathrm{YS}}$ is the yield strength, $\sigma_{0}$ and K are constants, and d is the average grain size [35].

It can be found from the above formula that there is an opposite relationship between the average particle size and yield strength. However, the mechanical properties are not only controlled by the size of grain but also the characteristics of eutectic phases and precipitates, as well as solid solubility of the Cu element. With increasing solid solubility, more Cu atoms were dissolved into Al matrix, definitely obstructing the movement of dislocations [36]. Coarse eutectic particles are easily subjected to stress concentration and act as preferred nucleation sites for cracks [37]. The reduction in the brittle, coarsening eutectic phase can improve the ductility of the large-scale ingot, reducing the chance for the formation of cracks during the deformation process [21].

## 5. Conclusions

In this paper, the influence of an ultrasonic field on the solidification process of Al-Cu alloys was systematically studied. Through experiments, the grain structure, eutectic morphology, and mechanical properties of the ingot were compared in the casting process with ultrasonic fields and conventional fields, and the eutectic phase was quantitatively analyzed. The following conclusions are drawn:

(1). An ultrasonic field is beneficial to refine the ingot grain. The grain-refinement efficiencies at the center, 1/2 radius, and edge of the ingot are 33.99%, 45.2%, and 41.68%, respectively, under the action of an ultrasonic field.

(2) Ultrasonics can refine the eutectic phase, especially the eutectic phase in the center of the ingot. The eutectic phase is a discontinuous dendrite without agglomeration.

(3) Ultrasonics can improve the solid solubility of alloying elements in ingots. Compared with conventional ingots, the solid solubility of Cu, Mn, Mg, and Si in ultrasonic ingots is improved, especially the solid solubility of Cu, which is increased by 67.05%.

(4) Ultrasonics can improve the dispersion degree of the eutectic phase in the ingot and then improve the mechanical properties of the material.

## Figures and Tables

**Table 1:** The chemical composition percentage of the Al-Cu alloy for the experiment (wt.%).

Composition	Si	Fe	Cu	Mn	Mg	Zn	Ti	Ni	Al
Content	0.93	0.10	4.37	0.78	0.63	<0.001	0.026	<0.001	Bal.

**Table 2:** Parameters of the eutectic phase calculated by the MATLAB program.

Sample	a-1	a-2	a-3	b-1	b-2	b-3
$V_{V}$	8.62%	8.92%	5.89%	4.34%	4.64%	4.76%
$P_{V}$(×10^4^/mm^3^)	3.57	3.27	2.72	1.57	1.83	1.64
λ(μm)	75	62	108	91	93	118

**Table 3:** Mass fraction of solute elements in crystal.

Element	Conventional Ingot (wt%)	Ultrasonic Ingot (wt%)	Increase Rate
Cu	0.85	1.42	67.05%
Mn	0.36	0.78	116.67%
Mg	0.52	0.60	14.56%
Si	0.07	0.28	300%

**Table 4:** Test results of tensile strength of tensile samples.

Number	Ultimate Tensile Strength of Ultrasonic Ingot (MPa)	Elongation of Ultrasonic Ingot (%)	Ultimate Tensile Strength of Conventional Ingot (MPa)	Elongation of Conventional Ingot (%)
Sample 1	165.26	1.72%	153.35	1.53%
Sample 2	170.94	2.09%	165.63	1.76%
Sample 3	192.39	3.80%	167.74	2.24%
Average value	176.20	2.54%	162.24	1.84%

## Data Availability

The data presented in this study are available on request from the corresponding author.
